# Effectiveness of pure argon for renal transplant preservation in a preclinical pig model of heterotopic autotransplantation

**DOI:** 10.1186/s12967-016-0795-y

**Published:** 2016-02-04

**Authors:** Alice Faure, Laurie Bruzzese, Jean-Guillaume Steinberg, Yves Jammes, Julia Torrents, Stephane V. Berdah, Emmanuelle Garnier, Tristan Legris, Anderson Loundou, Matthieu Chalopin, Guy Magalon, Regis Guieu, Emmanuel Fenouillet, Eric Lechevallier

**Affiliations:** Department of Urology and Kidney Transplantation, Aix-Marseille University, APHM, Marseille, France; UMR MD2 & IRBA, Aix-Marseille University, Marseille, France; Department of Pathology, Aix-Marseille University, APHM, Marseille, France; C.E.R.C, Aix-Marseille University, Faculty of Medicine, Marseille, France; Department of Nephrology and Kidney Transplantation, Aix-Marseille University, APHM, Marseille, France; Public Health Laboratory, Aix-Marseille University, Marseille, France; Air Liquide Medical Gases Group, Air Liquide sante International-Claude Delorme Research Center, Jouy-en-Josas, France; Department of Plastic Surgery, Aix-Marseille University, APHM, Marseille, France; Institut des Sciences Biologiques, CNRS, Paris, France

**Keywords:** Cold-storage solution, Organ preservation, Ischaemia–reperfusion injury, Kidney transplantation, Noble gas

## Abstract

**Background:**

In kidney transplantation, the conditions of organ preservation following removal influence function recovery. Current static preservation procedures are generally based on immersion in a cold-storage solution used under atmospheric air (approximately 78 kPa N2, 21 kPa O2, 1 kPa Ar). Research on static cold-preservation solutions has stalled, and modifying the gas composition of the storage medium for improving preservation was considered. Organoprotective strategies successfully used noble gases and we addressed here the effects of argon and xenon on graft preservation in an established preclinical pig model of autotransplantation.

**Methods:**

The preservation solution Celsior saturated with pure argon (Argon-Celsior) or xenon (Xenon-Celsior) at atmospheric pressure was tested versus Celsior saturated with atmospheric air (Air-Celsior). The left kidney was removed, and Air-Celsior (n = 8 pigs), Argon-Celsior (n = 8) or Xenon-Celsior (n = 6) was used at 4 °C to flush and store the transplant for 30 h, a duration that induced ischemic injury in our model when Air-Celsior was used. Heterotopic autotransplantation and contralateral nephrectomy were performed. Animals were followed for 21 days.

**Results:**

The use of Argon-Celsior vs. Air-Celsior: (1) improved function recovery as monitored via creatinine clearance, the fraction of excreted sodium and tubulopathy duration; (2) enabled diuresis recovery 2–3 days earlier; (3) improved survival (7/8 vs. 3/8 pigs survived at postoperative day-21); (4) decreased tubular necrosis, interstitial fibrosis, apoptosis and inflammation, and preserved tissue structures as observed after the natural death/euthanasia; (5) stimulated plasma antioxidant defences during the days following transplantation as shown by monitoring the “reduced ascorbic acid/thiobarbituric acid reactive substances” ratio and Hsp27 expression; (6) limited the inflammatory response as shown by expression of TNF-alpha, IL1-beta and IL6 as observed after the natural death/euthanasia. Conversely, Xenon-Celsior was detrimental, no animal surviving by day-8 in a context where functional recovery, renal tissue properties and the antioxidant and inflammation responses were significantly altered. Thus, the positive effects of argon were not attributable to the noble gases as a group.

**Conclusions:**

The saturation of Celsior with argon improved early functional recovery, graft quality and survival. Manipulating the gas composition of a preservation medium constitutes therefore a promising approach to improve preservation.

**Electronic supplementary material:**

The online version of this article (doi:10.1186/s12967-016-0795-y) contains supplementary material, which is available to authorized users.

## Background

Kidney transplantation is currently the preferred treatment for patients suffering from end-stage renal disease. The conditions used to preserve the organ following removal largely determine the short- and long-term function of the graft [1]. Kidney function recovery may be therefore considered as a marker of graft quality, and hence as a marker of the effectiveness of the graft storage procedure. Improvements in cold-storage conditions led to increased quality of the cadaveric kidney transplants although after transplantation a substantial percentage of grafts still do not function well. Delayed graft function (DGF) is a form of acute renal failure that occurs within the first week after transplantation. DGF, whose frequency is 2–50 %, associates in particular alteration in diuresis recovery with a need for dialysis, acute rejection episodes, and decreased mid/long-term survival [1–4]. DGF is mainly addressed via urine output but other traditional renal function parameters are also used, such as creatinine clearance (to address the glomerular filtration rate) and plasma urea (to address the renal excretory function). The main contributing factors to DGF include donor and recipient characteristics but also how the organ was treated prior to and following transplantation (e.g., prolonged cold-ischemia preservation with extended cold-ischemia time, prolonged warm ischemia) [3]. Over the past several years, ischemia–reperfusion injury (IRI) has been also considered as a major contributor to DGF, acute rejection, and chronic graft failure [1, 5]. Inherent in transplantation, IRI results from hypoxia and reoxygenation, and involves acidosis and cellular edema, cell damage, mitochondrial changes, oxidative stress, and inflammatory response [5]. Counteracting such detrimental mechanisms constitutes a challenge in transplantation and a major goal of the development of cold-storage preservation solutions. IRI is a complex process that is difficult to monitor with some studies having addressed the damage in the kidney context by monitoring acute tubular necrosis, interstitial fibrosis, apoptosis, and inflammation [3, 5].

Current static preservation procedures are based on immersion in a cold-storage solution under atmospheric air (approximately 78 kPa N2, 21 kPa O2, 1 kPa Ar). Research on cold-preservation solutions has stalled, and modifying the gas composition of the storage medium for improving preservation has been recently considered [6–8]. On one hand, oxygen has been used during hypothermic preservation in experimental and clinical models of kidney transplantation but the protective effects against organ injury are still in debate [9]. On the other hand, noble gases, e.g., argon and xenon, have been used to preserve perishable foods by limiting oxidation [10] prior to their development for graft preservation [6, 11]. Following this latter approach and using a rat model of kidney transplantation with a short cold-ischemia time (6 h), we reported that the use of the preservation solution Celsior saturated with pure argon (Argon-Celsior) improves graft functional recovery and limits IRI compared with the use of Celsior saturated with atmospheric air (Air-Celsior) [6].

Here, we further developed this approach in an established pig model of kidney autotransplantation. Although it is particularly difficult to implement, the pig model constitutes a significant improvement over the previous rat model because the size, anatomy, organization and physiology of the pig kidney are similar to those of its human counterpart, which makes it relevant for clinical practice [12, 13].

## Methods

### Animals

Female pigs (nourrain; 35 ± 2 kg; Les Crevoulins, France) were treated according to the University Guidelines of the Animal-Care Committee and housed in metabolic cages for blood- and urine-sampling.

### Surgical procedures

The pigs were prepared for surgery as previously described [14]. They were sedated using ketamine (30 mg/kg) and azaperone (1 mg/kg) via intramuscular injection followed by propofol (0.10–0.17 mg/kg/min) and remifentanyl (0.03–0.06 mg/kg/min) via a 20-gauge plastic catheter inserted into the auricular vein. Halothane was not used. Another catheter was placed in the jugular vein post-nephrectomy to monitor the biological parameters. Prior to left nephrectomy under sterile conditions (Additional file 1), 100 UI/kg of heparin sodium was injected intravenously. The transplant, including the vascular pedicle and ureter, was removed (warm ischemia <5 min) via midline laparotomy and flushed using the cold solution of interest prior to storage at 4 °C for 30 h (see below). Prior to transplantation, heparin sodium was administered again. The transplant was implanted by heterotopic autotransplantation via anastomosis of the renal vein and artery to the external right-iliac vessels. End-to-side uretero-ureteral anastomosis was performed without a stent. The transplant was immobilized by suturing the peritoneum. The contralateral kidney was removed to enable the monitoring of the function of the transplant only, and to serve as a control in the histological studies. The pigs were sacrificed at postoperative day-21 (POD21), chronic fibrosis occurring at this time in this model ([15] and data not shown). When death occurred before POD21 due to severe kidney dysfunction (surgical complications were always ruled out as a cause of death following autopsy), kidneys were taken within 0–3 h for analysis.

The animals were randomized into three groups: Air-Celsior (n = 8 animals; Air-Celsior animals thereafter), Argon-Celsior (n = 8; Argon-Celsior animals), and Xenon-Celsior (n = 6 for ethical reasons: see “Results” section; Xenon-Celsior animals). The characteristics of the animals and the surgical procedures were similar in all groups (Table 1).

**Table 1:** Characteristics of each pig group

	Experimental group		
	Air group (n = 8)	Argon group (n = 8)	Xenon group (n = 6)
Pig weight (kg)	35 ± 2	35 ± 2	35 ± 2
Kidney weight before preservation (g)	110 ± 2	111 ± 2	110 ± 1
Kidney weight after 30 h of preservation (g)	111 ± 2	110 ± 2	110 ± 0.5
Cold-ischemia time (h)	30 ± 0.3	30 ± 0.3	30 ± 0.2
Anastomotic times (min)			
Artery	24 ± 3	25 ± 4	24 ± 4
Vein	41 ± 4	42 ± 2	40 ± 2

### Saturation procedures and graft conditioning

**Fig. 1:**
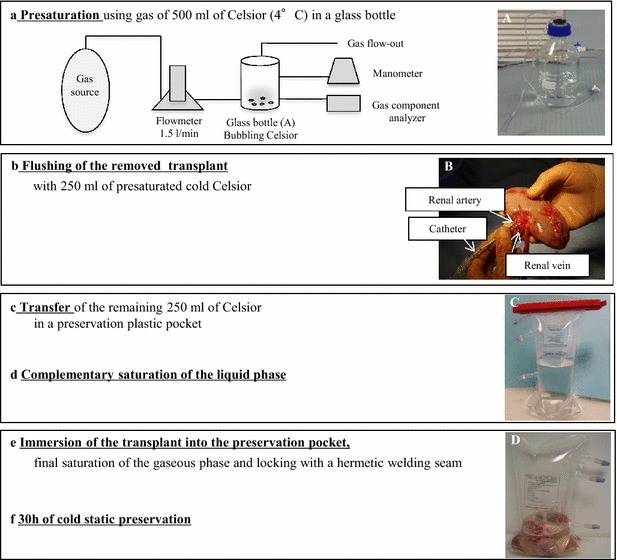
Saturation of Celsior with argon, xenon or atmospheric air. Celsior was saturated with argon, xenon or atmospheric air in a glass bottle (part *a*; picture **A**). The kidney graft was flushed using the cold solution of interest (250 ml) via a catheter inserted into the renal artery (*b*; **B**). The remaining solution (250 ml) was transferred in a plastic pocket (*c*; **C**) prior to an additional saturation step (*d*). The graft was then immersed in the solution of interest, and the pocket was sealed by welding (*e*; **D**). A final saturation step was performed prior to verifying that the atmosphere was appropriate. The graft was then stored for 30 h at 4 °C before transplantation (*f*)

Prior to left-sided nephrectomy, Celsior (Genzyme, France, 500 ml; 4 °C) was saturated with either atmospheric air (78 kPa N_2_, 21 kPa O_2_, 0.9 kPa Ar, assuming that the standard atmospheric pressure is 100 kPa; Air-Liquide Santé-France (Gentilly) or pure argon/xenon (100 kPa) in a laboratory glass bottle. The gas was injected into the bottle via connectors equipped with two cannulas (1.5 l/min; 5 min as determined in preliminary experiments), one cannula being immersed in the solution for bubbling, and the other one being used to equilibrate the pressure (Fig. 1). Measurements were performed in the gas phase using an electrochemical cell (Oxybaby 5.0; Wittgas, Germany) (n = 6–8 for each gas condition) and indicated that bubbling using pure argon or xenon led to a partial pressure of oxygen below the threshold of detection, the partial pressure in Air-Celsior being close to 21 kPa (i.e., 20 ± 0.8 kPa) when air was used for bubbling (the bubbling process tended to equilibrate partial pressure in the gas and liquid phases).

Immediately after removal, the transplant was flushed with the solution of interest (250 ml; 4 °C) via a catheter inserted into the renal artery. Flushing was stopped when a clear rinse liquid and transplant discolouration were observed. The kidney was weighed. The solution (250 ml; 4 °C) was also transferred in a plastic pocket equipped with three connectors (one in contact with the liquid phase, one in contact with the gas phase and one that allowed backpressure) and further saturated. The washed kidney was introduced, and the pocket sealed. A final saturation of the gas phase was performed, and the partial pressure of oxygen was confirmed (see above). Similar pressures were found again in a context of a collapsing pocket at the end of the storage time (30 h; 4 °C).

### Biological samples

Blood samples were collected prior to left nephrectomy and every day until POD7 and on POD9, POD14, and POD21. We monitored (UniCel-DxC800, Beckman-Coulter): (1) plasma (P_Cr_) and urinary (U_Cr_) creatinine to determine the creatinine clearance (C_Cr_), (2) plasma (P_u_) and urinary (U_u_) urea, and (3) plasma and urinary Na+ levels. We calculated: (1) the fraction of excreted sodium [FE_Na_; (urinary Na+/plasma Na+)/(urinary creatinine/plasma creatinine)], and (2) the urinary urea/plasma urea (U_u_/P_u_) ratio.

### Thiobarbituric acid reactive substances (TBARS) and reduced ascorbic acid (RAA)

Before left nephrectomy and at POD1, the oxidative stress was evaluated as previously described by addressing in the blood the lipid peroxidation process via TBARS and the antioxidant response via RAA, an increased RAA/TBARS ratio indicating an effective response to oxidative stress [16].

### Heat shock protein 27 (Hsp27)

Plasma Hsp27 level was quantified prior to left nephrectomy and every day until POD7, and on POD9, POD14 and POD21 as described using an ELISA kit (ab156498, Abcam, France; threshold: 0.1 ng/ml) [16]. All assays were performed in duplicate and the coefficient of variation was <5 %.

### TNF alpha-, interleukin-1 beta- and interleukin-6 assays

Kidney tissues were collected at POD21 post-euthanasia or within the first 3 h after death resulting from graft dysfunction. The level of expression of tumor necrosis factor alpha (TNF-alpha), interleukin-1 beta (IL1-beta) and interleukin-6 (IL6) was measured using ELISA kits (Uscn Life Science Inc.; E90133Po, E90563Po and E90079Po, with a detection limit <5.8 pg/ml, <5.4 pg/ml and <11.6 pg/ml for TNF-alpha, IL1-beta and IL6 respectively) according to the manufacturer’s instructions. All measurements were performed in duplicate, and the coefficient of variation was less than 5 %.

### Histological studies

The transplant was fixed using 4 % paraformaldehyde for 48 h. Wedge biopsies overlapping a region from the deep cortex to the outer-medulla region were collected at the end of cold storage or at POD21 post-euthanasia or within the first 3 h after death resulting from graft dysfunction. After paraffin embedding, tissue sections (5 micrometers) were stained using haematoxylin, eosin and safran. The degree of tubular lesions/necrosis (e.g., brush-border loss and endoluminal detachment) was scored as described [17] (1: no lesions; 2: lesions affecting <10 % of the sample; 3: 10–25 %; 4: 26–50 %; 5: >50 %). The interstitial inflammatory infiltrate was scored (1: no infiltrate; 2: loose, thin, dispersed infiltrate; 3: more abundant infiltrate, occasionally nodular; 4: dense, diffuse infiltrate with confluence). Fibrosis and tubular atrophy were scored (1: no fibrosis; 2: minimal fibrosis; 3: organized fibrosis with few glomerulosclerosis and tubular atrophy <25 %; 4: mutilating fibrosis with serious glomerulosclerosis and tubular atrophy >50 %). These observations were performed blind with respect to the graft treatment.

For immunohistochemical studies, kidney sections (5 mm) were incubated with a rabbit monoclonal antibody to the human active form of caspase-3 (BD Biosciences; clone C92-605; 1:200) or a mouse monoclonal to human CD10 (Diagnostic Biosystem; clone 56C6; 1:20) prior to labeling with the relevant staining system. Caspase-3 labeling was given as the percentage of cells expressing the antigen while CD10 labeling was determined using a semi-quantitative visual grading system as described [17] (−: no labeling; +: weak; ++: moderate; +++: strong). Five different tissue sections were analyzed. A LEICA DM 3000/DFC 452C equipment and the software program LEICA Application suite version 4.2.0 were used.

### Statistical analysis

The data are presented as the means ± the standard deviation (SD), or the medians with the interquartile ranges [IQRs]. Significant differences between groups were calculated using two-way multivariate ANOVAs with scheffe post hoc tests, Student’s tests or Kruskal–Wallis tests for multiple comparison analyses. A Kaplan–Meier survival analysis using a log-rank test was performed. p ≤ 0.05 was considered significant.

## Results

### Argon-Celsior improved early graft functional recovery and survival

A preservation time of 30 h was selected based on preliminary experiments. In contrast to renal transplantation in humans, the storage of pig kidneys in cold Air-Celsior for up to 24 h had no deleterious effects on graft function recovery and animal survival (data obtained in preliminary experiments and not shown). These data agree with those of a previous study that utilized a similar model [15]. We then tested increasing preservation times and found that a 30 h-storage significantly impaired graft function and survival, which constituted the criteria used to compare the effects of Argon-Celsior, Xenon-Celsior and Air-Celsior on transplant preservation.

**Fig. 2:**
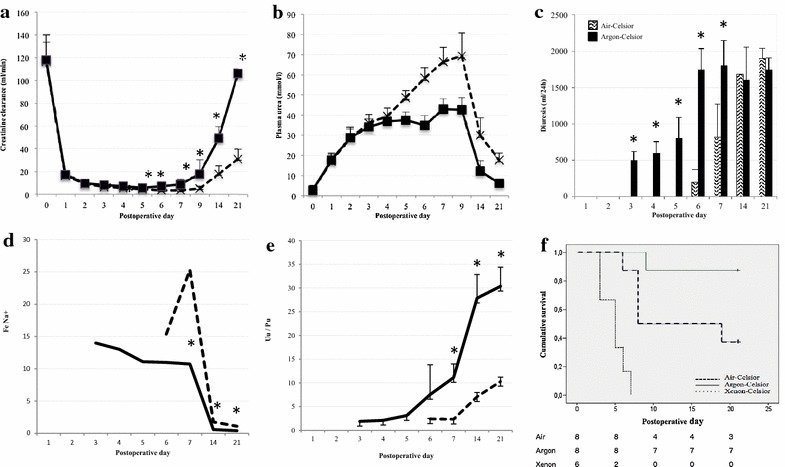
Argon-Celsior improved early graft functional recovery and survival. After transplantation, renal function was monitored using creatinine clearance (**a**; ml/min), plasma urea (**b**; mmol/l), diuresis (**c**; ml/24 h; diuresis before surgery: 1500–2000 ml/24 h), fraction of excreted sodium (FE Na + ; **d**) and the urine urea/plasma urea ratio (Uu/Pu; **e**). The data are reported as the means ± SDs; p < 0.050 (*asterisks*): Argon-Celsior vs. Air-Celsior. The survival data are plotted (**f**; *dashed line* Air-Celsior; *solid line* Argon-Celsior; *dotted line* Xenon-Celsior)

We treated accordingly kidney graft prior to implantation and monitored over 21 days the markers of kidney function in Argon-Celsior (n = 8) or Air-Celsior (controls; n = 8) animals. Creatinine clearance addresses the glomerular filtration rate of the kidney. The evolution of this parameter was similar in both groups prior to surgery (Argon-Celsior vs. Air-Celsior: mean ± SD: 116 ± 15 ml/min vs. 118.3 ± 16.5 ml/min) and decreased post-surgery (Fig. 2a). Compared with the controls, the Argon-Celsior animals exhibited: (1) a faster recovery of creatinine clearance [the function was considered to be efficient (*i.e.* > 60 ml/min) as soon as at POD14, reaching pre-transplant levels by POD21] and (2) a lower plasma urea value that reached a plateau at POD3 vs. a peak at POD7 (Fig. 2b; plasma urea addresses the renal excretory function).

Most Argon-Celsior animals (7/8) exhibited diuresis at POD7 vs. 3/8 Air-Celsior animals (Fig. 2c). The animals recovering diuresis survived until POD21. Diuresis recovery occurred significantly earlier in the Argon-Celsior group (median [IQR]: POD4 [3–5]; stable urine production began on POD6) compared to the controls (POD7 [6, 7]; stable urine production began on POD14). Compared to the controls, the Argon-Celsior animals exhibited: (1) a lower fraction of excreted sodium (Fig. 2d), and (2) an elevation in the urinary urea/plasma urea ratio beginning on POD6 (Fig. 2e), both variables addressing tubular function recovery. Thus, the Argon-Celsior group exhibited a shorter tubulopathy duration compared to the controls.

Regarding survival, 7/8 Argon-Celsior animals survived until POD21 and one animal died at POD9 following primary non-function (Fig. 2f). In contrast, only 3/8 Air-Celsior animals survived until POD21 and 5 animals died (POD8 [6–19]) of primary non-function. Thus, the use of Argon-Celsior improved survival compared to the use of Air-Celsior.

In parallel experiments, the use of Xenon-Celsior dramatically reduced survival: 4/6 pigs survived at POD4, 2/6 at POD6 and none at POD8 (Fig. 2f). The clinical status of the animals was dramatically altered as soon as POD4, with in particular low creatinine clearance (pre-operative values: 117 ± 20 ml/min; POD4: 3.10 ± 0.42 ml/min; POD6: 2.03 ± 0.51 ml/min) and high uraemia values (POD4: 52 ± 6 mmol/l; POD6: 68 ± 4 mmol/l). These data and the lack of urine production following transplantation by all six animals were indicative of acute renal failure. For ethical reasons, we therefore studied only six pigs. Additionally, the low survival rate (2/6 at POD6) prevented us from statistically analysing the variables used to monitor renal function. Together, the results obtained using xenon indicate that the positive effect of argon on pig survival was not attributable to the noble gases as a group.

### Storage in Argon-Celsior preserved the macroscopic appearance after reperfusion

**Fig. 3:**
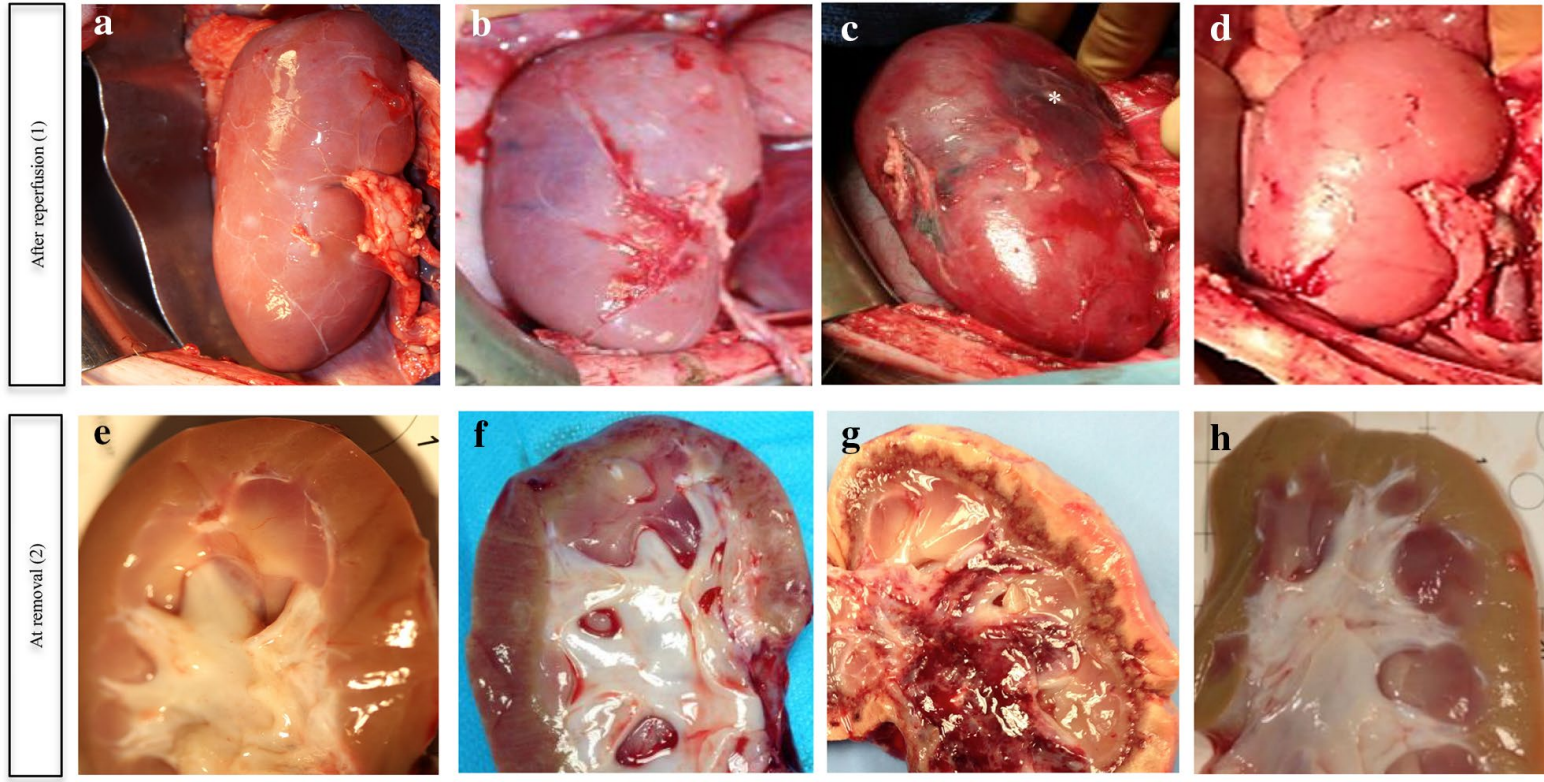
Argon-Celsior preserved transplant appearance. Per-operative pictures of the transplants 30 min after reperfusion (*1*) and at autopsy (*2*) are shown. A *right kidney* is shown and represents the authentic situation (control kidney; **a**, **e**). After reperfusion (*1*), macroscopic examination revealed major changes that included an impaired recolouration of the transplant in the Air-Celsior group (**b**) and further alterations in the Xenon-Celsior group (**c**) in addition to large necrotic areas (*asterisks*). The transplants stored in Argon-Celsior (**d**) exhibited appearances that were similar to those of the control kidneys. At autopsy (*2*), the Air-Celsior (**f**) and Xenon-Celsior (**g**) transplants exhibited significant cortical injuries compared with the Argon-Celsior transplants (**h**)

The macroscopic appearance of the grafts at the time of reperfusion was assessed (Fig. 3-1). The right kidney that was removed to enable the monitoring of the function of the transplant only was considered to represent the authentic appearance/structure (control organ; A). Large mottled red-blue areas with infarcts were observed when Air- (B) or Xenon-Celsior (C) was used, whereas the appearance of the grafts stored in Argon-Celsior (D) and that of the control organs were similar.

### Storage in Argon-Celsior preserved the tissue structure and the cell/epithelial integrity of the organ observed after the death of the animal

At autopsy (the autopsy was performed after the natural death of the animal or after euthanasia 21 days post-transplantation) (Fig. 3-2), the appearance of the kidneys taken from the Air-Celsior (F) or Xenon-Celsior (G) animals was poor (pale or with cortical mottled brown discolourations, respectively), whereas the organs from the Argon-Celsior (H) animals and the control organs had similar appearances.

**Fig. 4:**
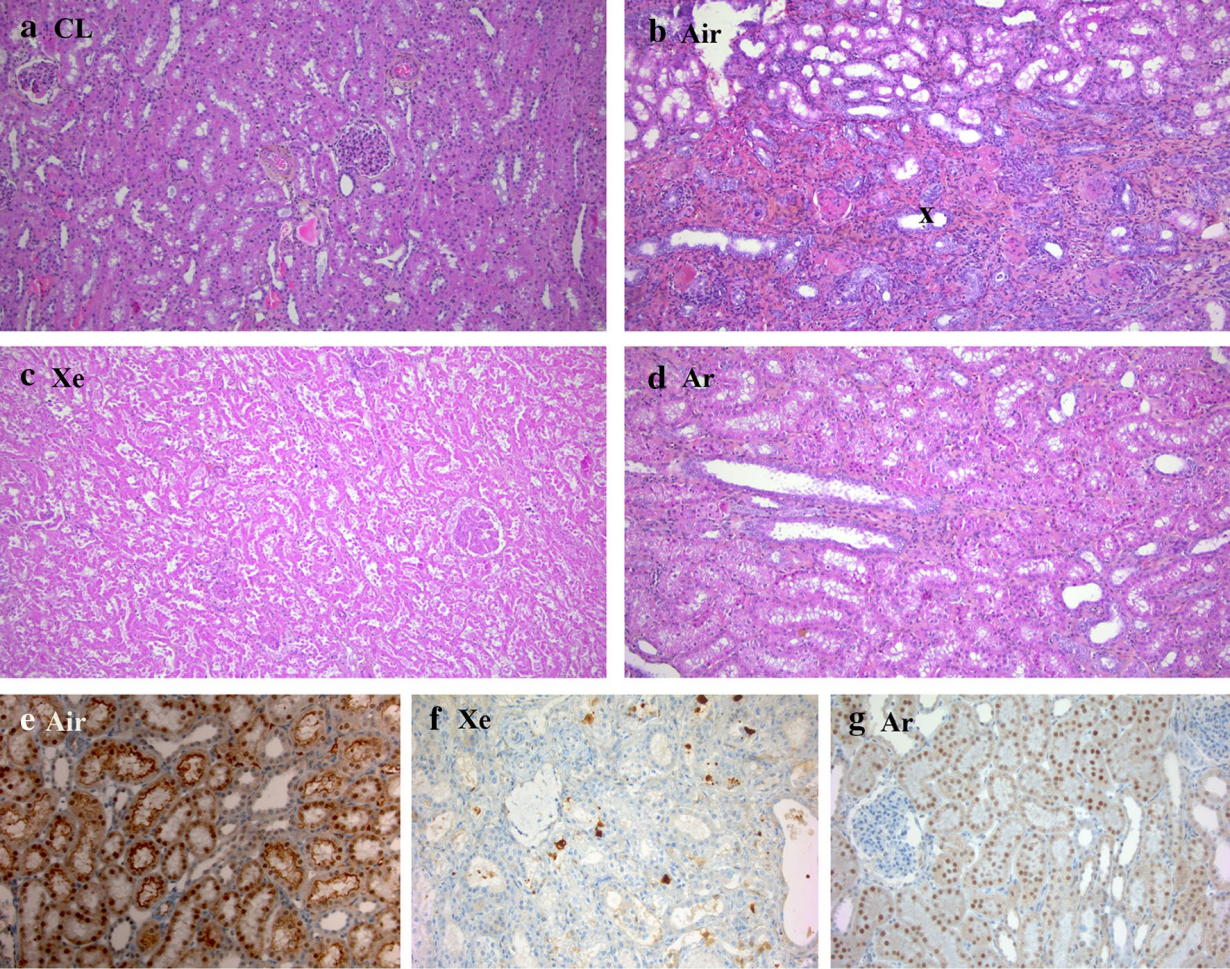
Argon-Celsior preserved tissue integrity. Light microscopy images of kidney tissue samples (×40). The contralateral kidney (see “Methods” section) was considered to represent the authentic renal architecture with normal tubules (CL; **a**). The kidney taken from the Air-Celsior animals (Air; **b**) exhibited tubule damage with tubular atrophy, interstitial fibrosis and lymphocyte infiltration (x). The kidney taken from the Xenon-Celsior animals (Xe; **c**) exhibited major lesions, including strong tubular atrophy and fibrosis. In contrast, the kidney taken from the Argon-Celsior animals (Ar; **d**) exhibited less tubule damage, weak tubular atrophy and satisfactory trophic signals. Immunostaining of caspase-3 expression associated with kidney samples taken at autopsy is shown (Air-Celsior group: Air; **e**; Xenon-Celsior group: Xe; **f**; ×40; Argon-Celsior group: Ar; **g**): labelling was weaker in Argon-Celsior transplants vs. Air-Celsior and no labelling was found in Xenon-Celsior transplants

Additionally, because the contralateral kidney (see above) represented the authentic renal architecture, we concluded from histological analysis following autopsy (Fig. 4a–d) that the tissue characteristics were better preserved in the Argon-Celsior than the Air-Celsior transplants with: (1) few dilated tubules, (2) effective tubule lumina, (3) discrete lesions of tubular necrosis, (4) lack of interstitial fibrosis, and (5) weaker tubular atrophy. Regarding tubular necrosis and atrophy, semi-quantitative scoring was performed and showed that cell- and epithelial-integrity was better preserved in the Argon-Celsior than the Air-Celsior transplants as observed at autopsy (Table 2). Conversely, no significant differences were observed in both groups at the end of the cold storage period when wedge biopsies were performed (Table 2). At autopsy again, and in contrast to the situation observed at the end of storage (Fig. 4a; Table 2), the lymphocyte infiltration was less pronounced in the Argon-Celsior transplants, indicating less inflammation. In contrast, the tissue structure was altered in the Xenon-Celsior transplants (Fig. 4c; Table 2).

**Table 2:** Histological study of the graft at various time points

Condition	Time points	Histology			Immunolabeling	
		Tubular lesions	Lymphocyte infiltration	Fibrosis and tubular atrophy	Caspase-3 (%)	CD10
Air-Celsior	Removal	2.2 ± 0.1	1	1	3	+++
	End of storage	2.3 ± 0.1	1	1	5	+++
	Autopsy	4.7 ± 0.2	1.8 ± 0.2	2.7 ± 0.1	40	+
Argon-Celsior	Removal	2.1 ± 0.1	1	1	3	+++
	End of storage	2.2 ± 0.1	1	1	5	+++
	Autopsy	3.3 ± 0.1*	1.2 ± 0.1	1.3 ± 0.1*	24	+++
Xenon-Celsior	Removal	2.0 ± 0.1	1	1	4	+++
	End of storage	2.2 ± 0.1	1	1	6	+++
	Autopsy	4.6 ± 0.1	1.9 ± 0.1	2.6 ± 0.2	0	−

Tubular lesions (1: no lesions; 2: lesions affecting <10 % of the sample; 3: 10–25 %; 4: 26–50 %; 5: >50 %), lymphocyte infiltration (1: no infiltrate; 2: loose, thin, dispersed infiltrate; 3: more abundant infiltrate, occasionally nodular; 4: dense, diffuse infiltrate with confluence), and fibrosis and tubular atrophy were scored (1: no fibrosis; 2: minimal fibrosis; 3: organized fibrosis with few glomerulosclerosis and tubular atrophy <25 %; 4: mutilating fibrosis with serious glomerulosclerosis and tubular atrophy >50 %). Data are means ± SDs; p < 0.050 (*): Argon-Celsior vs. Air-Celsior. Caspase-3 labeling was given as the percentage of cells expressing the antigen. CD10-labeling was determined using a semi-quantitative visual grading system (−: no labeling; +: weak; ++: moderate; +++: strong)

The active form of caspase-3 is immunodetected in the renal cortex during apoptosis whereas caspase-3 labeling is usually below the threshold of detection in control conditions. As expected, we observed very little tissue labeling in the contralateral kidney (authentic situation). Similar findings were obtained at the end of storage in the three groups whereas at autopsy, caspase-3 labeling was weaker in Argon-Celsior transplants compared with Air-Celsior transplants and no labeling was found in the Xenon-Celsior group (Fig. 4e–g; Table 2). These results are consistent with caspase-3 activation following apoptosis during the post-transplantation course in a context of moderate cell damage (here, in the Argon-Celsior group and even more significantly in the Air-Celsior group), its expression level becoming undetectable as cell disorganization and necrosis progress (here, in the Xenon-Celsior group) [18]. Additionally, we used CD10 labeling to demonstrate the integrity of the proximal tubular epithelial cell, in particular the tubular brush border. Labeling was readily observed in the contralateral kidneys and, at the end of storage and at autopsy, in Argon-Celsior transplants. In contrast, CD10 expression was weak at autopsy in Air-Celsior transplants and below the threshold in Xenon-Celsior transplants (Table 2).

Together, these results indicate that storage in Argon-Celsior improved the subsequent fate of the cellular and tissue structures of the transplant vs. Air-Celsior, Xenon-Celsior being strongly detrimental.

### Argon improved the antioxidant response and limited inflammation

In the following experiments, we monitored: (1) RAA/TBARS ratio and Hsp27 expression to address the antioxidant response in a short and mid-term perspective, respectively [16, 19], and (2) TNF-alpha, IL1-beta and IL6-expression to evaluate inflammation.

**Fig. 5:**
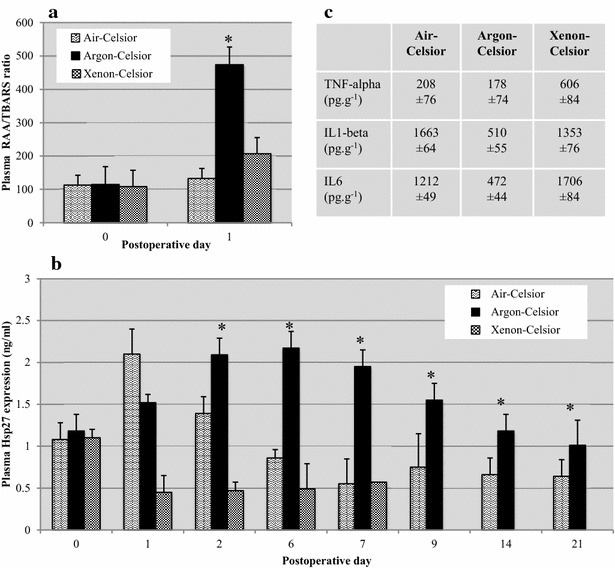
Argon-Celsior stimulated antioxidant defences and limited inflammation. Before left nephrectomy and at postoperative day 1, the antioxidant status was assessed via plasma RAA/TBARS ratio (**a**; *RAA* reduced acorbic acid; *TBARS* thiobarbituric acid reactive substances) and plasma expression of Hsp27 (**b**). Tissue expression of TNF-alpha, Interleukin-1 beta and Interleukin-6 was determined (**c**). The data are reported as the means ± SDs; p < 0.050 (*asterisks*): Argon-Celsior versus Air-Celsior and p < 0.050 (*bash*): Argon-Celsior versus Xenon-Celsior

Early after reperfusion, the RAA/TBARS ratio significantly increased in the Argon-Celsior group vs. the Air-/Xenon-Celsior groups (473.9 ± 77.9, 132.8 ± 47.9, 206.8 ± 51.1, respectively; Fig. 5), which indicates that the antioxidant response was promoted early after reperfusion when argon was used. Plasma expression of Hsp27 increased day after day during the first week after transplantation when argon was used, reaching a peak at POD6, whereas Hsp27 expression was significantly weaker from POD2 in the Air-/Xenon-Celsior groups. Analysis of Hsp 27 expression using the Scheffe’s post hoc test indicated however a lack of significant difference (Air-group vs Argon-group: mean difference = 0.75, p value = 0.371 with 95 % confidence interval (−0.70;2.2); Air-group vs Xenon-group: mean difference = 0.05, p value = 0.989 with 95 % confidence interval (−0.99;1.10); Argon-group vs Xenon-group: mean difference = −0.69, p value = 0. 405 with 95 % confidence interval (−2.11;0.72)). Finally, TNF-alpha was strongly produced when xenon was used instead of air or argon whereas IL1-beta and IL6 expression was weaker in the Argon-Celsior group vs. the Air-/Xenon-Celsior groups. These data give mechanistic insights into how argon improved graft preservation, indicating that the gas limited oxidative injury and inflammation, and promoted antioxidant defence.

## Discussion

While argon was found to exert a protective effect on myocardial/cerebral ischemia and traumatic brain injury [11, 20–23], the protective effect of this gas in renal transplantation is poorly documented [6, 11, 24]. In this study, using a relevant preclinical pig model of renal autotransplantation, we found that the saturation of the extracellular preservation solution with pure argon had beneficial effects on early graft functional recovery and survival compared with the use of atmospheric air. In contrast, the use of xenon was dramatically detrimental.

At the tissue and molecular level, and when analysis was performed after the natural death of the animal or 21 days post transplantation, we observed that argon significantly improved tissue structure preservation by decreasing tubular necrosis, interstitial fibrosis, apoptosis, and inflammation. Because these histological processes are hallmarks of IRI [3, 5], we propose that argon is effective to limit deleterious consequences that are considered to be generated during IRI and renal transplantation. Of note however, the tissue and molecular characteristics were found to be rather similar in the three groups at the end of the cold storage period, suggesting that most of the effects of argon we observed in this study developed following transplantation. These latter observations are consistent with the fact that the cold storage period almost completely inhibits cell metabolism.

We also addressed after transplantation some of the molecular mechanisms that may explain the influence of argon on tissue preservation. The heat shock protein Hsp27 is involved in signal transduction and has antioxidant, antiapoptotic, antinecrotic and cytoprotective properties [19, 25, 26]. We therefore considered that its monitoring may give clues about how kidney preservation was improved by argon. We observed that Hsp27 expression increased following transplantation in Air-/Argon-Celsior animals. Two days post reperfusion however, it is noteworthy that its expression still grew only when Argon was used during storage. These data suggest that argon exerts its protective effect at least partly by upregulating Hsp27 expression. These results are in agreement with previous reports that showed that Hsp27 expression provides a strong survival advantage in a context of redox stress and inflammation, in particular by stimulating the antioxidant defenses of the cell [26]. Our present data are consistent with a previous study [27] and support the idea that increased Hsp27 expression constitutes an adaptive response to control IRI.

The increased Hsp27 expression when Argon-Celsior was used is consistent with the observation that argon prevents apoptosis [28] and attenuates IRI [29] inasmuch as these two mechanisms are partly driven by Hsp27 via cellular signaling and regulation of several functions such as the maintenance of mitochondrial integrity [22, 26]. The beneficial effect of the increased expression of Hsp27 when argon was used was probably supported in the animals by the increased RAA/TBARS ratio, a result that indicates that the response to the redox injury was promoted.

In this study, the use of xenon adversely affected graft functional recovery and survival outcome. Expression of the markers of the oxidative injury (RAA/TBARS ratio and Hsp27 expression after transplantation) and inflammation (TNF-alpha/Interleukin-1beta/Interleukin-6 expression at autopsy) was in full agreement with the observations made at the tissue level in the corresponding animals and with the fate of the animals post-transplantation. The opposite effects that were induced by argon and xenon indicate that the effects of argon are not attributable to the noble gases as a group and that the substitution of oxygen and/or nitrogen by another gas is not sufficient. The detrimental effect of xenon observed here contrasts with previous reports [6, 20, 23, 28–31], but it is notable that these latter used experimental conditions that differed in the following manners. Firstly, small (e.g. rats) vs. large (pigs in our study) animals: the gas can quickly diffuse in/out of a kidney of weak volume (rat) contrary to the situation encountered using a large animal (pig) in which the diffusion distance is considerably higher. Secondly, gas inhalation vs. organ storage in a modified gas environment, as utilized here, gas exchange by lungs in blood being different from bubbling in Celsior. Thirdly, a mixture of gas and oxygen vs. pure gas, as used here. Finally, the inability of xenon to protect the graft in hypoxic conditions as observed here may be related to the fact that its diffusion coefficient is lower than that of argon in kidney [32], which influences the gas exchange process that occurs during storage, and following transplantation and blood flow restoration. Performing oxygen gradient analysis within the transplant using a tissue oxygen monitoring cell would be interesting to determine oxygen concentration after storage and oxygen diffusion after reperfusion, two processes that may explain the discrepant effects induced by argon and xenon. Conversely, we confirmed here previous studies indicating that argon lacks adverse effects, even when pure [6, 11].

There are three main limitations to our study. First, we used Celsior because it is a widely used cold-storage solution that elicits no differences in post-transplantation outcomes relative to the University of Wisconsin (UW) or histidine–tryptophan–ketoglutarate (HTK) solutions [33, 34]. Further investigations are needed however, to examine whether our findings can be reproduced using solutions other than Celsior and alternative preservation methods (e.g., pulsatile perfusion). Secondly, we did not explore the dose-response relationship between argon concentration and survival, and the effect of saturation of the preservation solution using mixtures of argon, nitrogen and oxygen at various concentrations needs to be addressed. Thirdly, the effect of argon during the preservation period per se was found to be discrete as wedge biopsies performed at the end of storage did not enable to detect histological differences among the three groups. The exact time where argon triggered its effect during the procedure remains therefore unclear.

## Conclusions

Saturation of Celsior with pure argon improved early graft functional recovery and survival, and affected markers associated with IRI pathways. Thus, the manipulation of the gas component of a preservation medium—and in particular the use of argon to saturate the storage solution—constitutes a promising approach to improving transplant preservation in clinical conditions.

## Additional file

10.1186/s12967-016-0795-y The surgical technique of heterotopic renal autotransplantation in pigs.

## Abbreviations

C_Cr_: creatinine clearance
DGF: delayed graft function
FE_Na_: fraction of excreted sodium
Hsp27: heat Shock Protein 27
HTK: histidine–tryptophan–ketoglutarate solution
IL1-beta: interleukin-1 beta
IL6: interleukin-6
IRI: ischemia-reperfusion injury
P_Cr_: plasma creatinine
P_u_: plasma urea
POD: postoperative day
U_Cr_: urinary creatinine
U_u_: urinary urea
U_u_/P_u_: urinary urea/plasma urea ratio
UW: University of Wisconsin solution
RAA: reduced ascorbic acid
TBARS: thiobarbituric acid reactive substances
TNF-alpha: tumor necrosis factor alpha

## References

[1] Quiroga I, McShane P, Koo DD, Gray D, Friend PJ, Fuggle S (2006). Major effects of delayed graft function and cold ischaemia time on renal allograft survival. Nephrol Dial Transplant.

[2] Ojo AO, Wolfe RA, Held P, Port FK, Schmouder RL (1997). Delayed graft function: risk factors and implications for renal allograft survival. Transplantation.

[3] Perico N, Cattaneo D, Sayegh MH, Remuzzi G (2004). Delayed graft function in kidney transplantation. Lancet.

[4] Koning OHJ, Ploeg RJ, Van Bockel JH, Groenewegen M, van der Woude FJ, Persijn GG (1997). Risk factors for delayed graft function in cadaveric kidney transplantation: a prospective study of renal function and graft survival after preservation with University of Wisconsin solution in multi-organ donors. European Multicenter Study Group. Transplantation.

[5] Wilhelm MJ, Pratschke J, Laskowski I, Tilney NL (2003). Ischemia and reperfusion injury. Transplant Rev.

[6] Irani Y, Pype JL, Martin AR, Chong CF, Daniel L, Gaudart J (2011). Noble gas (argon and xenon)-saturated cold storage solutions reduce ischemia-reperfusion injury in a rat model of renal transplantation. Nephron Extra.

[7] Abe T, Li XK, Yazawa K, Hatayama N, Xie L, Sato B (2012). Hydrogen-rich University of Wisconsin solution attenuates renal cold ischemia-reperfusion injury. Transplantation.

[8] Ozaki KS, Yoshida J, Ueki S, Pettigrew GL, Ghonem N, Sico RM (2012). Carbon monoxide inhibits apoptosis during cold storage and protects kidney grafts donated after cardiac death. Transpl Int.

[9] Hosgood SA, Nicholson HF, Nicholson ML (2012). Oxygenated kidney preservation techniques. Transplantation.

[10] Buchheit RG, Schreiner HR, Doebbler GF (1966). Growth responses of neurospora crassa to increased partial pressures of the noble gases and nitrogen. J Bacteriol.

[11] Coburn M, Sanders RD, Ma D, Fries M, Rex S, Magalon G (2012). Argon: the ‘lazy’ noble gas with organoprotective properties. Eur J Anaesthesiol.

[12] Bagetti Filho HJ, Pereira-Sampaio MA, Favorito LA, Sampaio FJ (2008). Pig kidney: anatomical relationships between the renal venous arrangement and the kidney collecting system. J Urol.

[13] Dehoux JP, Gianello P (2007). The importance of large animal models in transplantation. Front Biosci.

[14] Faure A, Maurin C, Bruzzese L, Rolland PH, Coulange C, Pype J (2013). An experimental porcine model of heterotopic renal autotransplantation. Transplant Proc.

[15] Cavallari G, Catena F, Santoni B, Montalti R, Turi P, Beltempo P (2002). Kidney preservation in pigs with University of Wisconsin and Celsior solution. Minerva Chir.

[16] Jammes Y, Steinberg JG, Delliaux S (2012). Chronic fatigue syndrome: acute infection and history of physical activity affect resting levels and response to exercise of plasma oxidant/antioxidant status and heat shock proteins. J Intern Med.

[17] Goujon JM, Hauet T, Menet E, Levillain P, Babin P, Carretier M (1999). Histological evaluation of proximal tubule cell injury in isolated perfused pig kidneys exposed to cold ischemia. J Surg Res.

[18] Yang B, Nahas AM, Thomas GL, Haylor JL, Watson PF, Wagner B (2001). Caspase-3 and apoptosis in experimental chronic renal scarring. Kidney Int.

[19] Arrigo AP (2005). In search of the molecular mechanism by which small stress proteins counteract apoptosis during cellular differentiation. J Cell Biochem.

[20] Jawad N, Rizvi M, Gu J, Adevi O, Tao G, Maze M (2009). Neuroprotection (and lack of neuroprotection) afforded by a series of noble gases in an in vitro model of neuronal injury. Neurosci Lett.

[21] Ryang YM, Fahlenkamp AV, Rossaint R, Wesp D, Loetscher PD, Beyer C (2011). Neuroprotective effects of argon in an in vivo model of transient middle cerebral artery occlusion in rats. Crit Care Med.

[22] Fahlenkamp AV, Rossaint R, Haase H, Al Kassam H, Ryang YM, Beyer C (2012). The noble gas argon modifies extracellular signal-regulated kinase 1/2 signalling in neurons and glial cells. Eur J Pharmacol.

[23] Pagel PS, Krolikowski JG, Shim YH, Venkatapuram S, Kersten JR, Weihrauch D (2007). Noble gases without anesthetic properties protect myocardium against infarction by activating prosurvival signaling kinases and inhibiting mitochondrial permeability transition in vivo. Anesth Analg.

[24] Rizvi M, Jawad N, Li Y, Vizcaychipi MP, Maze M, Ma D (2010). Effect of noble gases on oxygen and glucose deprived injury in human tubular kidney cells. Exp Biol Med.

[25] Vidyasagar A, Wilson NA, Djamali A (2012). Heat shock protein 27 (HSP27): biomarker of disease and therapeutic target. Fibrogenesis Tissue Repair.

[26] Arrigo AP, Virot S, Chaufour S, Firdaus W, Kretz-Remy C, Diaz-Latoud C (2005). Hsp27 consolidates intracellular redox homeostasis by upholding glutathione in its reduced form and by decreasing iron intracellular levels. Antioxid Redox Signal.

[27] Guo Q, Du X, Zhao Y, Zhang D, Yue L, Wang Z (2014). Ischemic postconditioning prevents renal ischemia reperfusion injury through the induction of heat shock proteins in rats. Mol Med Rep.

[28] Ulbrich F, Kaufmann KB, Coburn M, Lagrèze WA, Roesslein M, Biermann J (2015). Neuroprotective effects of Argon are mediated via ERK-1/2 dependent regulation of heme-oxygenase-1 in retinal ganglion cells. J Neurochem.

[29] Spaggiari S (2013). Antiapoptotic activity of argon and xenon. Cell Cycle.

[30] Zhao H, Luo X, Zhou Z, Liu J, Tralau-Stewart C, George AJ (2014). Early treatment with xenon protects against the cold ischemia associated with chronic allograft nephropathy in rats. Kidney Int.

[31] Ma D, Hossain M, Pettet GK, Luo Y, Lim T, Akimov S (2006). Xenon preconditioning reduces brain damage from neonatal asphyxia in rats. J Cereb Blood Flow Metab.

[32] Warr O, Ballentine CJ, Mu J, Masters A (2015). Optimizing Noble Gas-Water Interactions via Monte Carlo Simulations. J Phys Chem B.

[33] O’Callaghan JM, Knight SR, Morgan RD, Morris PJ (2012). Preservation solutions for static cold storage of kidney allografts: a systematic review and meta-analysis. Am J Transplant.

[34] Parsons RF, Guarrera JV (2014). Preservation solutions for static cold storage of abdominal allografts: which is the best?. Curr Opin Organ Transplant.

